# Aqueous Two-Phase Systems Based on Cationic and Anionic Surfactants Mixture for Rapid Extraction and Colorimetric Determination of Synthetic Food Dyes

**DOI:** 10.3390/s23073519

**Published:** 2023-03-28

**Authors:** Svetlana V. Smirnova, Vladimir V. Apyari

**Affiliations:** Department of Chemistry, Lomonosov Moscow State University, Moscow 119991, Russia; sv_v_smirnova@mail.ru

**Keywords:** colorimetric determination, aqueous two-phase systems, benzethonium chloride, preconcentration, food dyes, food analysis, UV-Vis spectrophotometry

## Abstract

In this study, aqueous two-phase systems (ATPSs) containing a cationic and anionic surfactants mixture were used for the preconcentration of the synthetic food dyes Allura Red AC, Azorubine, Sunset Yellow, Tartrazine, and Fast Green FCF. A rapid, simple, low cost, affordable, and environmentally friendly methodology based on microextraction in ATPSs, followed by spectrophotometric/colorimetric determination of the dyes, is proposed. The ATPSs are formed in mixtures of benzethonium chloride (BztCl) and sodium N-lauroylsarcosinate (NaLS) or sodium dihexylsulfosuccinate (NaDHSS) under the molar ratio close to equimolar at the total surfactant concentration of 0.01–0.20 M. The density, viscosity, polarity, and water content in the surfactant-rich phases at an equimolar ratio BztCl:NaA were determined. The effects of pH, total surfactant concentration, dye concentration, and time of extraction/centrifugation were investigated, and the optimum conditions for the quantitative extraction of dyes were established. The smartphone-based colorimetric determination was employed directly in the extract without separating the aqueous phase. The analytical performance (calibration linearity, precision, limits of detection and quantification, reproducibility, and preconcentration factor) and comparison of the spectrophotometric and smartphone-based colorimetric determination of dyes were evaluated. The method was applied to the determination of dyes in food samples and food-processing industrial wastewater.

## 1. Introduction

Synthetic food dyes have become widespread in various fields of the food and pharmaceutic industries. They are extensively used to impart desired colors to foodstuffs, drinks, and pharmaceutical formulations [1,2]. In spite of their relatively low toxicity, intake of food colorants in large amounts can cause some negative effects on human health [3,4,5]. Another problem is contamination with the dyes of different environmental objects, which may result in negative impacts on natural ecosystems [6,7].

Both these problems make the development of effective analytical methods for the determination of synthetic food dyes at different concentration levels a genuine concern [8]. Today, a wide variety of such methods have been proposed, such as electrochemical methods [9,10,11,12], colorimetry [13,14,15], spectrophotometry [16,17], fluorimetry [18,19], and high-performance liquid chromatography [20,21,22]. However, in many cases, the most logical and environmentally friendly approach to determine the dyes without the use of additional reagents and organic solvents is one using their own absorption, i.e., a colorimetric approach [23,24].

Colorimetry is a modern, rapidly developed method that is very compatible with such widespread devices as smartphones, digital cameras, and scanners and easily implemented in out-of-laboratory analysis [25,26,27,28]. At the same time, there are some problems for this method, mainly associated with its relatively low sensitivity and the need for sample preparation to avoid interference from matrix components.

Different sample preparation methods, including mainly solid-phase and liquid-phase extraction, have been developed to extract and preconcentrate food dyes [29,30]. In recent times, a prominent trend can be identified in this field, aiming to make the sample preparation techniques more suitable for the concept of “green″ analytical chemistry. Form this viewpoint, good prospects reveal such extractants as aqueous two-phase systems (ATPSs) or aqueous biphasic systems (ABSs). These systems are obtained by mixing two different water-soluble components, such as polymers, alcohols, salts, or ionic liquids, which become immiscible under certain temperature conditions, pressure, pH, or concentration [31,32]. In addition to the well-known polymer/polymer, polymer/salt, and salt/salt ATPSs, aqueous mixtures of some cationic–anionic surfactants can separate spontaneously into two immiscible aqueous phases [33]. One phase is poor in surfactant and is called the dilute surfactant phase. The other phase contains a high concentration of surfactant and is called the surfactant-rich or coacervate phase [34]. This phenomenon, caused by a change in concentration, occurs at high concentrations of surfactants, depending on their nature, structure, and molar ratio. However, the limitation of these mixed systems is their tendency to form crystalline precipitates in an aqueous solution as a result of the coulombic interaction between oppositely charged species [35]. It was shown that the ionic nature of the surfactant polar head groups, the length of the hydrocarbon chain, and its branching and asymmetry significantly influence the phase behavior of the cationic/anionic surfactant aqueous solution [36,37]. The phase separated from a large sample volume may have a small volume, so high preconcentration coefficients can be achieved. The volume ratios of the two phases depend on the concentrations and molar ratios of the two surfactants. ATPSs have high water content and provide easy phase transition for highly water-soluble hydrophilic compounds that are difficult to extract in conventional systems. ATPSs have been successfully applied to separate and preconcentrate various species, such as biomaterials, dyes, organic compounds, and metal ions [38,39,40,41]. Such systems are very promising for the extraction of common water-soluble, synthetic, sulfonated dyes.

The components of ATPSs are commercially available and are generally more environmentally friendly and less expensive surfactants, compared with some organic salts. In this work, we studied ATPSs based on the cationic surfactant, benzethonium chloride (BztCl), which has antiseptic and anti-infective properties and is used in detergents, softeners, and cosmetics, as well as in the food industry as a disinfectant for surfaces. In addition, the benzethonium cation is known as a component of some ionic liquids, an ion-associated phase in two-phase systems, and some eutectic solvents [42,43,44]. The anionic surfactants were sodium N-lauroylsarcosinate (NaLS) and sodium dihexylsulfosuccinate (NaDHSS). NaLS is widely used as a detergent, foaming agent, and cleaning agent. NaDHSS is used as an emulsifier, dispersant, solubilizer, and wetting agent. In addition, hydrophobic ionic liquids containing LS^-^ and DHSS^-^ anions are known and investigated as extractants [45,46].

NaLS and NaDHHS are micelle-forming surfactants, with critical micelle concentrations (CMCs) equal to 14.57 and 14.1 mmol L^−1^, respectively [47,48]. BztCl can form micelles at lower concentrations; its CMC is 0.4 mmol L^−1^ [47]. The aggregation in aqueous mixtures of cationic and anionic surfactants occurs at considerably lower concentrations than the CMC of the individual surfactants [49]. A significant decrease of the CMC of the surfactants can be explained by the formation of vesicles, along with mixed micelles containing a 1:1 ion-pair complex. Thus, a strong anionic–cationic synergy between the hydrocarbon chains of the constituent surfactants leads to a lower CMC value [50]. When solutions of cationic and anionic surfactants are mixed, the strong reduction in area per head group due to ion pairing causes the formation of molecular bilayers at low concentrations [49]. Mixtures of cationic and anionic surfactants in aqueous solutions can self-assemble into various microstructures, such as spherical micelles, rodlike micelles, vesicles, etc. In general, planar bilayer structures, such as vesicles, often form. The solubilization capacity of such aggregate structures may be higher than that of spherical micelles [34]. This is a very beneficial characteristic for extraction. The solutes can be simultaneously extracted and preconcentrated in the surfactant-rich phase, depending on the similarity in their polarity.

In this paper, within the concept of “green″ analytical chemistry, we propose novel aqueous two-phase systems based on a BztCl and NaLS/NaDHSS mixture that can be effectively applied for rapid extraction and following the colorimetric determination of synthetic food dyes directly in the extract with a smartphone.

## 2. Materials and Methods

### 2.1. Reagents and Instruments

Benzethonium chloride, BztCl (97%, Acros Organics, Geel, Belgium); sodium dihexyl sulfosuccinate, NaDHSS (Technolog Ltd., Moscow, Russia); sodium N-lauroylsarcosinate, NaLS (98%, Sigma, St. Louis, MO, USA); 2,6-diphenyl-4-(2,4,6-triphenylpyridin-1-yl)-phenolate monohydrate (Reichardt’s dye, Sigma–Aldrich, 70%); and ethanol (95%, Ekros, Russia) were used as supplied. The synthetic dyes Azorubine (≥50%, Aldrich, St. Louis, MO, USA), Allura Red AC (≥80%, Sigma-Aldrich, St. Louis, MO, USA), Fast Green FCF (≥85%, Sigma-Aldrich), Sunset Yellow (90%, Aldrich, Delhi, India), and Tartrazine (≥85%, Sigma, USA), as well as hydrochloric acid and sodium hydroxide (98%, Panreac, Darmstadt, Germany), were used as supplied.

Solutions containing 0.1464 mol·L^−1^ of BztCl, NaLS, and NaDHSS were prepared by dissolving a weighed amount of solid salts in high-purity water. Stock solutions of dyes (2.0·10^−2^ mol L^−1^) were prepared by dissolving proper amounts of the dyes in distilled water. All working solutions were prepared daily by diluting the stock solution in distilled water. Hydrochloric acid (2 mol L^−1^) and sodium hydroxide (0.5 mol L^−1^) were used for pH adjustments. All other reagents employed in this work were of analytical grade.

Absorbance measurements were performed using a U-2900 double-beam UV–VIS spectrophotometer (Hitachi, Tokyo, Japan); the samples were placed into quartz micro-cuvettes (Hellma GmbH & Co. KG, Müllheim, Germany) with a light path of 1 cm. A model 410 pH meter (Akvilon, Russia) with a combined glass microelectrode ELSK-13.7 was used for pH measurements. Centrifugation required for faster phase separation was performed on a Hettich EBA-20 centrifuge (Tuttlingen, Germany). Chemicals were weighed using a ViBRA HT (Shinko Denshi, Tokyo, Japan) analytical balance. The measurement of water content in the surfactant-rich phase was made using an 870 KF Titrino plus titrator (Metrohm, Herisau, Switzerland). The viscosity measurements were carried out on a Brookfield viscometer CAP 2000+H (USA) equipped with four cone-and-plate geometry and using a Peltier system for controlling the temperature.

An iPhone 11 smartphone equipped with a Dual 12 MP camera system (Apple Inc., Cupertino, CA, USA) was used to take photos of the samples. To obtain RGB values from the photos, the free access application RGBer iOS App (Version 2.6) was run on the iPhone. Freeware programs built into the iPhone were used for photo adjustment and image analysis.

### 2.2. Phase Diagram Construction

#### 2.2.1. BztCl–NaLS–H_2_O

A phase diagram was constructed to determine the total concentrations and the molar ratio of BztCl and NaLS at which the phase separation occurs. The concentration range of phase-forming components was selected in accordance with the amount of separating organic phase deemed convenient for microextraction. The surfactant stock solutions of 0.2 mol L^−1^ BztCl and NaLS were prepared by dissolving weighed amounts of the surfactants in pure water. ATPSs were prepared by mixing the stock solutions at a molar ratio of 1:1, but having different total concentrations of components at 21 ± 2 °C. Further, the samples were titrated drop-wise with the solution of one of the components until the opalescence caused by the emulsification of one phase into another disappeared and the system turned clear, i.e., until one phase was formed [51]. The content of the components at these points corresponds to the points on the binodal curve. The data for constructing the BztCl–NaLS–H_2_O diagram are presented in Table S1.

#### 2.2.2. BztCl–NaDHSS–H_2_O

Constructing a phase diagram for the BztCl–NaDHSS–H_2_O system is complicated by the very limited region of the molar ratio around the equimolar and narrow concentration range, corresponding to the existence of the two-phase liquid–liquid system, as well as the presence of a wide concentration range, in which one of the phases is solid. Therefore, titration does not allow visually detecting the transition between the two-phase liquid–liquid state and the states in which the solid phase is present in the system. To determine the liquid–liquid phase separation boundaries of the anionic and cationic surfactant mixtures, a series of solutions with a molar ratio of BztCl:NaDHSS = 1:1, but with a variable total surfactant concentration (*c*_t_), as well as a series of solutions with a constant concentration of NaDHSS and a varying concentration of BztCl, were prepared [47,52]. The various surfactant concentrations were obtained by mixing the stock solutions in different ratios. The samples were kept at room temperature (21 ± 2 °C) for at least one week, until equilibrium was reached. Phase equilibrium is achieved when a clear interphase boundary forms. The presence of two liquid phases was determined visually. The samples in which two independent clear liquid phases coexisted were classified as ATPSs. The data for constructing the BztCl–NaDHSS–H_2_O diagram are given in Table S2.

### 2.3. Procedure

#### 2.3.1. Extraction in ATPS Systems BztCl–NaDHSS–H_2_O

A 10.0 mL portion of sample solution containing a dye was placed in a 15 mL polypropylene centrifuge tube at room temperature (21 ± 2 °C). If necessary, the pH of the aqueous phase was adjusted with 2.0 mol L^−1^ HCl and 0.5 mol L^−1^ NaOH. Then, 0.5 mL of 0.1464 mol L^−1^ BztCl and 0.5 mL of 0.1464 mol L^−1^ NaDHSS were added successively, resulting in cloudy solutions. The samples were stirred manually for 5 s and placed in hot water (85 ± 5 °C) for 30 s to ensure the rapid separation of the liquid surfactant-rich phase from the water. In order to speed up phase separation, the cloudy solutions were centrifuged for 10 min at 6000 rpm.

#### 2.3.2. Extraction in ATPS Systems BztCl–NaLS–H_2_O

A 10.0 mL portion of sample solution containing a dye was placed in a 15 mL polypropylene centrifuge tube at room temperature (21 ± 2 °C). If necessary, the pH of the aqueous phase was adjusted with 2.0 mol L^−1^ HCl and 0.5 mol L^−1^ NaOH. Then, 0.8 mL of 0.1464 mol L^−1^ BztCl, and 0.8 mL of 0.1464 mol L^−1^ NaLS were added successively, forming cloudy solutions. The samples were stirred manually for 5 s and centrifuged for 10 min at 6000 rpm.

The extraction efficiency (*E*, %) of dyes was calculated as:

$$E\left(\%\right)=100\cdot \left(1-\frac{c_{w}V_{w}}{c_{w}^{0}V_{w}^{0}}\right)$$

where $c_{w}^{0}$ and $c_{w}$ are the initial and final (equilibrium) concentrations (mg L^−1^) of a dye in the aqueous phase, respectively; $V_{w}^{0}$ and $V_{w}$ are the initial and final volumes (mL) of the aqueous phase, respectively. All experiments were performed three times, and the standard deviation of the results did not exceed 5% (unless otherwise indicated by error bars).

#### 2.3.3. Colorimetric Determination of Dyes after Extraction in ATPSs

**Scheme 1:**
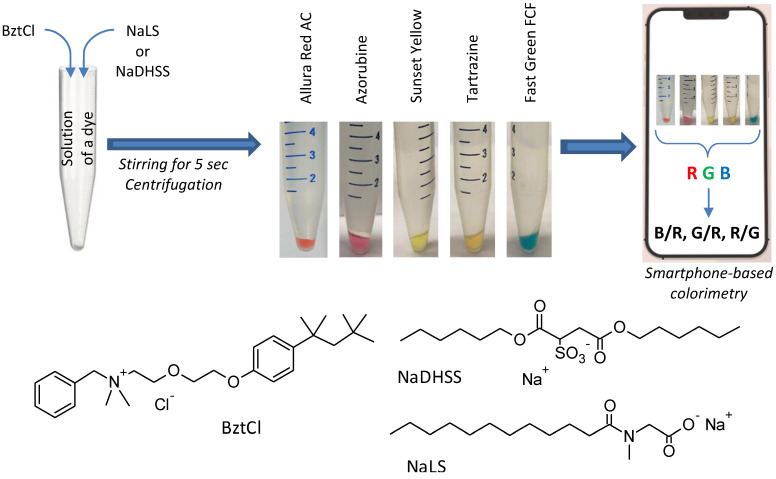
Principle of the colorimetric determination of dyes after preconcentration in benzethonium-based ATPSs.

Digital image acquisition was performed directly at the tube, without removing the upper aqueous phase. The tubes were fixed vertically in a tripod holder on a white background to maintain the same ambient light and photographic conditions. In addition to the usual laboratory lighting, a 50 W white LED spotlight (SDO-5 series pro) with a high light intensity was used. The smartphone was placed parallel to the test tubes, pointing the camera at the bottom of the conical tube with the extract, in such a way as to ensure that measurements were always carried out in the same place. The distance between the tube with ATPS and the smartphone camera was approximately 15 cm. Digital image measurements were performed for at least 10 replicates for every sample. The intensity values of the red–green–blue (RGB) color coordinates of the lower phase were estimated using a smartphone application that converts images to RGB values (Scheme 1).

#### 2.3.4. Spectrophotometric Determination of Dyes after Extraction in ATPSs

The upper aqueous phase after centrifugation was poured into a clean test tube, and the equilibrium pH values were measured. The extracts (lower surfactant-rich phases containing the dyes) remaining at the bottom of the tube were diluted with 0.4 mL of ethanol. The content of dyes in the diluted lower (surfactant-rich) and upper (surfactant-depleted) phases was determined by UV–Vis spectroscopy. The absorbance was measured at 509, 520, 485, 430, and 620 nm for Allura Red AC, Azorubine, Sunset Yellow, Tartrazine, and Fast Green FCF, respectively, against distilled water. To construct calibration curves, 10.0 mL of the solution containing a proper concentration of dyes was used. The same procedure was applied for the blank solution.

### 2.4. Sample Preparation

Soft drinks containing Allura Red AC (“Bubble″, Kaskad, Belarus) and Azorubine (“Atom″, Darida, Belarus), marked on their packages, were purchased from local supermarkets. The determination of dyes in beverages was carried out without additional sample preparation steps. A 1.0 mL aliquot of beverage was transferred into a 100 mL volumetric flask and diluted with distilled water. Finally, the developed methods were applied to the 10.0 mL aliquot of the diluted drink.

The pharmaceutical drug “Nitroxalin″ (Biosintez, Russia) containing Sunset Yellow in the shell was purchased from local suppliers. Its stock solution was prepared according to the following procedure: 10.0 mL of distilled water was added to one whole tablet. The solution was separated by decantation after the dissolution of the shell. After twenty times dilution, the proposed method was applied.

Food-processing industrial wastewater (Moscow “Udarnitsa″ factory, Russia) was collected in a plastic polyethylene bottle. The water samples were kept in the dark at 4 °C until the analysis. The developed preconcentration procedure was directly applied to the wastewater, followed by the colorimetric and spectrophotometric determination of dye contents. Finally, the spiked samples at each concentration were analyzed in triplicate with the developed procedures.

## 3. Results and Discussion

### 3.1. Formation and Properties of ATPSs Based on Benzethonium Chloride

**Figure 1:**
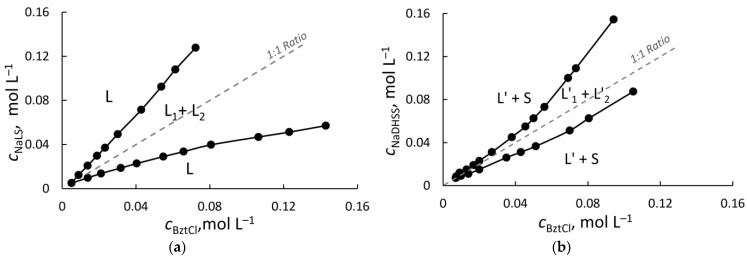
Phase diagram for benzethonium-based ATPSs (21 ± 2 °C): (**a**) BztCl–NaLS–H_2_O; (**b**) BztCl– NaDHSS–H_2_O.

The phase behavior of mixed cationic and anionic surfactants solutions near the equimolar composition was evaluated for the surfactants’ ability to form aqueous two-phase systems. The phase behavior of the BztCl–NaLS–H_2_O mixtures at different mixing ratios and concentrations was first investigated. Figure 1a shows the phase diagram for the BztCl–NaLS–H_2_O at 22 °C. All concentrations are presented in molar units (mol L^−1^). The total surfactants concentration (*c*_t_) was 0.0015–0.2 mol·L^−1^ and the molar ratios of BztCl to NaLS were 0.57–2.5 mol/mol. It was established that there are two regions, monophasic (L) and the ATPS region (L_1_ + L_2_), depending on the BztCl/NaLS molar ratio. The diluted mixtures (*c*_t_ < 0.005 mol·L^−1^) of BztCl and NaLS at an equimolar ratio produced a clear solution. With an increase in the total concentration of the surfactants, turbidity appeared. Yet, at *c*_t_ ≥ 0.01 M, two immiscible liquid phases formed. The homogeneous phase and ATPS were observed when the total surfactant concentration was higher than 0.01 mol·L^−1^. The mixtures of BztCl and NaLS formed a clear solution at a BztCl/NaLS molar ratio lower than 0.57 and above 2.5, that is, at *x_BztCl_* < 0.36 and at *x_BztCl_* > 0.65 (L region in Figure 1a). Two immiscible liquid phases were obtained at the total surfactant concentration region of 0.010–0.2 mol·L^−1^ with 0.36 ≤ *x_BztCl_* ≤ 0.65. With an increase in the total surfactant concentration, the two-phase region expanded significantly. Both liquid phases were transparent mobile liquids. The bottom phase was surfactant-rich, and the top phase was surfactant-depleted. There was a clear interface between the two immiscible water phases in the L_1_ + L_2_ area, and the interface between the two phases was quickly restored after remixing. The system BztCl–NaLS–H_2_O displayed a large biphasic area compared to similar known aqueous two-phase cationic/anionic surfactant systems [33,39,41].

The pH of the spontaneously formed system when mixing aqueous solutions of BztCl and NaLS was 6.5 ± 0.2. The formation of ATPS occurred at pH ≥ 6. At pH values below 5.5 ± 0.5, the BztCl–NaLS–H_2_O system was a viscous emulsion resistant to centrifugation. This is consistent with the usual p*K*_a_ values in the range of 5.0–7.0 for carboxylate surfactants, which are the sodium salts of fatty acids and N–acyl amino acids, when dissolved in micelles of other surfactants [50,53]. Protonation of the COO^-^ headgroup of NaLS leads to the weakening of the interaction between the oppositely charged head groups of surfactants and prevents the formation of ATPS. Then, extraction in this system was investigated at pH ≥ 6. The ratio of the upper and lower phase volumes did not depend on a pH within the range of 6.0–12.5.

The phase diagram for BztCl–NaDHSS–H_2_O is obtained by mixing aqueous solutions of BztCl and NaDHSS (Figure 1b). ATPS (region L′_1_ + L′_2_) is formed at the surfactants total concentration of 0.012–0.2 mol·L^−1^ with 0.45 ≤ *x*_BztCl_ ≤ 0.55. The upper phase is the surfactant diluted phase, and the lower phase is the surfactant-rich phase. For diluted mixtures of surfactants at *c*_t_ < 0.012 mol·L^−1^, as well as at a BztCl/NaDHSS molar ratio below 0.71 and above 1.33 mol/mol, a heterogeneous system with a precipitate is formed (region L + S). Previously, the precipitation phase boundaries in the BztCl/NaDHSS mixture in the presence of 0.15 mol·L^−1^ sodium chloride at 23 °C was established [47], but the existence of a two-phase liquid–liquid system in the surfactant mixtures was not reported. The formation of a two-phase system is limited by a relatively narrow range of surfactant concentrations. The fine formation conditions require an accurate BztCl/NaDHSS ratio to obtain ATPS. When mixing surfactant solutions, a milk-like emulsion formed. Phase separation was required from 30 min to 24 h, depending on the surfactant concentration. With an increasing surfactant concentration, the time required for spontaneous phase separation was decreased. Centrifugation for 10 min at 6000 rpm provided phase separation. For diluted mixtures of the surfactants, the tubes with the cloudy solution were first placed in hot water (85 ± 5 °C) for 30 s. ATPS has a clear interfacial boundary at the equimolar NaDHSS/BztCl ratio in the pH range from 1.0 to12.0. The ratio of phase volumes does not depend on pH.

Characterization of the systems obtained with the equimolar ratio of BztCl and NaA (where A = LS or DHSS) was performed in more detail at the total surfactant concentration of 0.073 mol·L^−1^. Mixing of aqueous solutions of BztCl and NaA in a ratio of 1:1 (1 mL of 0.1464 M BztCl and 1 mL of 0.1464 M NaA were added to 2.0 mL of water) resulted in the formation of ATPSs. At the same time, the volumes of the lower and the upper phases were 0.20 ± 0.05 mL and 3.9 ± 0.05 mL, respectively, for the BztCl/NaLS/H_2_O system and 0.12 ± 0.05 mL and 3.9 ± 0.05 mL, respectively, for the BztCl/NaDHSS/H_2_O system. Obviously, high *V*_upper_:*V*_lower_ ratios are beneficial for an analytical preconcentration (supposing that the lower layer is a concentrate). The phases enriched with surfactants were viscous transparent liquids with a density higher than that of water, which was 1.0112 and 1.1920 g cm^−3^ (25 °C) for BztCl–NaLS–H_2_O and BztCl–NaDHSS–H_2_O, respectively. Their viscosities were 0.1766 and 2.167 Pa∙s (25 °C) for BztCl–NaLS–H_2_O and BztCl–NaDHSS–H_2_O, respectively. A relatively high viscosity of the surfactant-rich phase does not affect the mass transfer rate and extraction efficiency. Since a fine dispersion of the extractive phase occurs at the moment of its formation, the large surface area of the contacting phases provides high extraction efficiency. The viscosity of the surfactant-rich benzethonium-based system is comparable to or exceeds the viscosity of some similar ATPSs and polymer-based ABSs [33,41,54,55,56].

We evaluated the polarity of the surfactant-rich phases using the solvatochromic response of Reichardt’s betaine dye [57]. The Dimroth–Reichardt polarity parameters E_T_(30) were equal to 50.9 and 48.6 kcal mol^−1^ for BztCl–NaLS–H_2_O and BztCl–NaDHSS–H_2_O, respectively. These values are close to the polarity of such alcohols as 1-propanol (50.7) and 1-octanol (48.3) [57]. In this regard, a good solubilizing/extracting ability of the surfactant-rich phases is expected towards highly hydrophilic compounds.

Both systems are characterized by high water content in the surfactant-rich phase. The measured water content was 68% wt. (or 0.98 molar fractions, χ = 98%) for BztCl–NaLS–H_2_O and 13.4% wt. (or 0.87 molar fractions, χ = 87%) for BztCl–NaDHSS–H_2_O. High water content in both immiscible phases is typical for ATPSs. This provides a favorable water-like environment for the extraction of highly hydrated high-polar/ionic compounds, for which extraction with conventional solvents is ineffective. The evaluated water content for benzethonium-based systems is comparable to those for ATPSs based on water-soluble polymers and organic salts [45,58,59,60].

### 3.2. Optimization of Dye Extraction in Benzethonium-Based Systems

The extraction of dyes in benzethonium-based ATPSs was studied and optimized. Extraction was carried out for each dye separately. The residual concentration of dyes after extraction in the upper aqueous phase was determined by UV–Vis spectroscopy.

#### 3.2.1. Effect of pH

The studied dyes exist in the aqueous solutions as the anionic forms in a wide pH range due to the presence of sulfo groups in their structure. The extraction of ionizable compounds is known to depend significantly on the ionic state. The extraction of 5 dyes was studied within the pH ranges of 1.2–11.5 for BztCl–NaDHSS–H_2_O and 6.6–11.5 for BztCl–NaLS–H_2_O.

**Figure 2:**
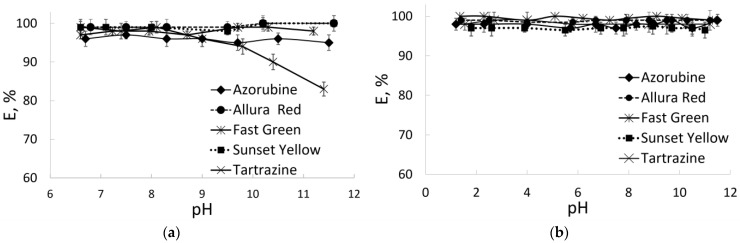
Effect of pH on dye extraction in benzethonium-based ATPSs: (**a**) BztCl–NaLS–H_2_O; *c*_BztCl_ = *c*_NaLS_ = 0.02 mol L^−1^ (**b**) BztCl– NaDHSS–H_2_O; *c*_BztCl_ = *c*_NaDHSS_ = 0.02 mol L^−1^. Dye concentration: Allura Red, Azorubine, Sunset Yellow, Tartrazine 20 mg L^−1^; Fast Green, 5 mg L^−1^.

As can be seen from Figure 2, all dyes were extracted quantitatively. The extraction efficiency remained constant over the investigated pH range. A similar lack of pH effect on dye extraction was observed earlier for a number of aqueous two-phase systems, including those based on organic salts [16,45]. The high water content in the extracting phase, much more than 0.5 mole fractions, provides a favorable environment for ionized hydrophilic compounds. The transfer of extracted species to a phase with high water content, apparently, is carried out without their dehydration. It is also important that the extracting phase has an ionic character, and the electrostatic attraction between the dye anions and benzethonium cations can promote their extraction.

Water-soluble synthetic food dyes are poorly extracted into conventional solvents, except for alcohols. It is noteworthy that the polarity of low-molecular-weight alcohols is close to the polarity of the surfactant-based phases. Thus, the polarity of the solvent is one of the key factors determining its extraction ability. For further work, dye extraction was performed without adjusting the pH from about 6.5–7.2.

#### 3.2.2. Effect of Total Surfactant Concentration on Extraction Efficiency and Phase Ratio

**Figure 3:**
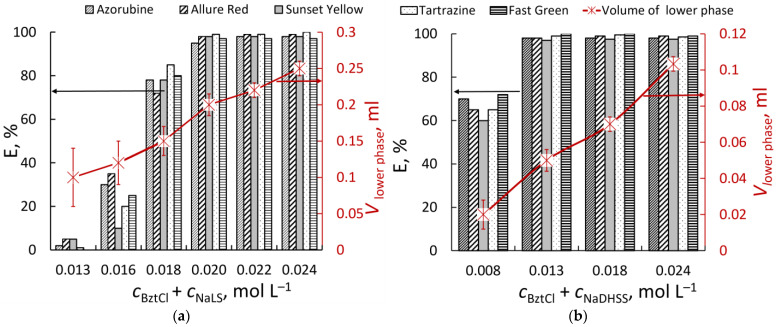
Effect of total surfactant concentration on dye extraction in benzethonium-based ATPSs: (**a**) BztCl–NaLS–H_2_O; (**b**) BztCl– NaDHSS–H_2_O. Dye concentration: Allura Red, Azorubine, Sunset Yellow, Tartrazine 20 mg L^−1^; Fast Green, 5 mg L^−1^, pH 6.5–7.5.

A phase separation of benzethonium-based systems depends on the total concentration of surfactants *c*_t_ and their molar ratio. Extraction in both systems was carried out at an equimolar ratio BztCl/NaA (where A = LS or DHSS), since this provided a longer range of total concentration of ATPS formation. The total concentration was varied within the range of ATPS existence. With an increase in *c*_t_, the volume of the separating surfactant-rich phase increased. It was noted that the presence of analytes facilitated the formation of ATPS. The separation of the surfactant-rich phase occurred at a lower total *c*_t_ than in their absence. Figure 3 shows the dependence of the extraction efficiency and the volume of the lower phase on the total surfactant concentration. All studied dyes were extracted quantitatively in BztCl–NaDHSS–H_2_O at *c*_t_ ≥ 0.013 mol L^−1^. The volume of the separated phase was 55 ± 5 μL. The preconcentration factor calculated as the phase volume ratio was 194. The extraction efficiency for all dyes was more than 95% at *c*_t_ exceeding 0.020 mol L^−1^ in BztCl–NaLS–H_2_O. The volume of the lower phase was 200 ± 10 μL, and the preconcentration factor was 57. At a concentration equal to 0.018 mol L^−1^ and below, the extraction efficiency did not exceed 50%. At a concentration < 0.015 mol L^−1^, the formation of a two-phase system was unstable and characterized by low reproducibility of the lower phase volume. Accordingly, *c*_t_ = 0.013 mol L^−1^ was selected for further study of BztCl–NaDHSS–H_2_O and 0.020 mol L^−1^ for BztCl–NaLS–H_2_O.

#### 3.2.3. Effect of Salt Addition

The addition of salts can influence liquid–liquid phase separation and the extraction efficiency due to the salting-out effect. The amount of sodium chloride was investigated in the range of 0–10% (*w*/*v*). It was found that the presence of salt does not affect the phase behavior. The extraction of dyes is quantitative. At a content of 10%, a slight decrease in the volume of the separated phase of 5–10% from the initial one was observed.

#### 3.2.4. Effect of Shaking and Centrifugation Time

The effect of shaking and the centrifugation time on the extraction efficiency was evaluated. The shaking time, studied over the range of 0–60 s, had no effect on the extraction efficiency, since extraction occurs simultaneously with the formation of the extracting phase. The smallest droplets of the dispersed phase have a large surface area, which ensures fast mass transfer and efficient extraction.

Centrifugation is a critical step to separate the surfactant-rich phase. The centrifugation time was varied from 2 to 30 min at 6000 rpm. The study showed that phase separation was achieved within 10 min of centrifugation for BztCl–NaDHSS–H_2_O and 5 min for BztCl–NaLS–H_2_O ATPS.

### 3.3. Analytical Performance

The analytical performance (calibration range, precision, limits of detection (LODs) and quantitation (LOQs), reproducibility, preconcentration factor, and other characteristics) was estimated under optimal conditions: volume of sample solution 10.0 mL; total surfactant concentration 0.02 and 0.013 mol L^−1^ for BztCl–NaDHSS–H_2_O and BztCl–NaLS–H_2_O, respectively; and equimolar ratio BztCl/NaA (where A = LS or DHSS).

To select a color channel that provides maximum sensitivity, the dependencies of the R, G, and B color coordinates on the concentration were plotted for each dye. The color channel that allows for maximum sensitivity was selected. The strongest decrease in the detected light intensity with the increasing concentration of Allura Red AC, Sunset Yellow, and Tartrazine was found for the B channel. The G channel had the maximum sensitivity for Azorubine, and the R channel for Fast Green FCF. To eliminate the effect of variable illumination, the ratio of the most to the least sensitive color coordinate was considered as an analytical signal. The use of relative color coordinates made it possible to increase the precision of the measurements by several times.

The RGB values for the surfactant-rich phase were recorded at various concentrations of each dye, as well as in the absence of a dye (a blank sample). Calibration curves were constructed with 10 concentration levels, using the relative color coordinate B/R (for Allura Red AC, Sunset Yellow, and Tartrazine), G/R (for Azorubine), or R/G (for Fast Green FCF).

In contrast to the absorbance, which is proportional to the concentration of a colored compound, the color coordinates are linearly related to the intensity of recorded radiation, which can be calculated as *I* = *I*_0_∙*exp(−klc).* This presumes an exponential type of the color coordinate-based calibration curves. Good correlations between the B/R, G/R, R/G ratios and the concentration were found when fitted with the exponential functions *Y* = *Y*_0_ + *A*_1_·*exp*(*c*/*t*_1_), where *Y* the is value of the B/R, G/R, or R/G ratio, *Y*_0_, *A*_1_, and *t*_1_ are parameters of the exponential function, and *c* is the concentration of a dye. In the low concentration region, the relationship between the relative color coordinates and the concentration is linear for Sunset Yellow, Tartrazine, and Fast Green FCF. In comparison with linear data approximation, exponential fitting allows expanding the determination range and increasing the sensitivity. For all dyes, the colorimetric determination is possible directly in the extract, without its separation, using a smartphone. The squared correlation coefficients (*R*^2^) were 0.9928–0.9985. The limit of detection and quantitation were calculated from 3 and 10 standard deviations, respectively, for 10 consecutive measurements of the blank to the slope of the calibration curve *A*_1_/*t*_1_.

**Figure 4:**
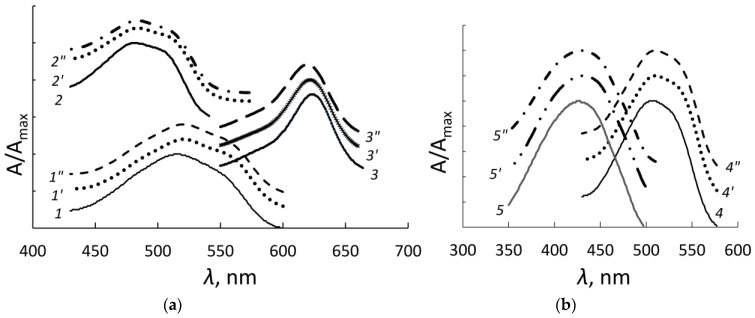
The absorption spectra of aqueous solutions of dyes (1–5) and diluted extracts obtained in BztCl–NaDHSS –H_2_O (1′–5′) and BztCl–NaLS –H_2_O (1″–5″) ATPSs: (**a**) Azorubine (1, 1′, 1″), Sunset Yellow (2, 2′, 2″), Fast Green FCF (3, 3′, 3″); (**b**) Allura Red AC (4, 4′, 4″), Tartrazine (5, 5′, 5″). Dye concentration: Allura Red, 1.7 mg L^−1^; Azorubine, 1.3 mg L^−1^; Sunset Yellow, Tartrazine, 1.8 mg L^−1^; Fast Green, 0.73 mg L^−1^. pH 6.5–7.5. For dilution of the extracts: *V*ethanol = 0.4 mL.

The analytical characteristics obtained for the smartphone-based procedure were compared with those obtained by conventional spectrophotometric determination. The measurements were carried out at the wavelengths of the absorption maxima (509, 520, 485, 430, and 620 nm for Allura Red AC, Azorubine, Sunset Yellow, Tartrazine, and Fast Green FCF, respectively) after preconcentration in APTSs (Table 1). The absorption spectra of aqueous solutions of dyes before extraction and the absorption spectra of the diluted extracts obtained in BztCl–NaA–H_2_O ATPSs (where A = LS or DHSS) are shown in Figure 4. The shape of the spectra and absorption maxima for aqueous solutions and extracts for each dye were close to each other. A slight shift in the absorption maximum for extracts can be explained by a change in the polarity of the solvent.

In general, the determination range for the colorimetric method is narrower compared to spectrophotometry. Compared with the spectrophotometric determination, the smartphone-based method has a detection limit 2–5 times higher for all dyes after preconcentration in BztCl–NaLS–H_2_O and for Allura Red AC and Fast Green FCF after preconcentration in BztCl–NaDHSS-H_2_O. For BztCl–NaDHSS–H_2_O, the LODs of Azorubine, Sunset Yellow, and Tartrazine are comparable for both methods. Colorimetric determination using BztCl–NaDHSS–H_2_O is generally more sensitive than using BztCl–NaLS–H_2_O (LODs are 2–6 times lower in the first case).

The relative standard deviation was calculated for 3 replicates at 0.35 mg L^−1^ Allura Red, Azorubine, Sunset Yellow, and Tartrazine and 0.08 mg L^−1^ Fast Green FCF. Good accuracy values were obtained in the ranges of 3–12% and 1–6% for the colorimetric and spectrophotometric determination, respectively. The preconcentration factor was 194 for BztCl–NaDHSS-H_2_O (V_lower_ = 55 ± 5 μL, V_upper_ = 10.7 ± 1.0 mL) and 57 for BztCl–NaLS–H_2_O (V_lower_ = 200 ± 10 μL, V_upper_ = 11.3 ± 1.0 mL). The obtained results demonstrate that the smartphone-based method provides suitable and precise measurements.

The proposed procedure has several advantages. The colorimetric determination after the preconcentration procedure is carried out in a single step, without the need for the removal of the aqueous phase and the dilution of the surfactant-rich phase by ethanol used in the spectrophotometric measurements. Reducing the number of steps involved in sample processing improves the accuracy and recovery. Direct colorimetric determination reduces the sample preparation time and provides a high preconcentration factor. Another advantage of the proposed technique is the cheapness and availability of the phase-forming components, their low consumption due to the high phase volume ratio achieved.

### 3.4. Interference Studies

The effects of potentially interfering substances, such as citric acid, sugar, ascorbic acid, phenylalanine, glutamic acid, and Na^+^, K^+^, Cl^−^, SO_4_^2−^, on the extraction of Allure Red AC in benzethonium-based ATPSs under optimal conditions were examined. Variation over ±10% in the analytical signal that resulted from foreign ions was considered as interference. It was demonstrated that the determination of 0.4 mg L^−1^ Allure Red AC after preconcentration in BztCl–NaDHSS-H_2_O is not affected, at least by 1000-fold quantities of sucrose, ascorbic acid, and citric acid and 100-fold quantities of phenylalanine, glutamic acid, Na^+^, K^+^, Cl, and SO_4_^2−^. The determination of the dye in BztCl–NaLS–H_2_O is not affected by at least 1000-fold quantities of sucrose and ascorbic acid and 100-fold quantities of citric acid, phenylalanine, glutamic acid, Na^+^, K^+^, Cl^−^, and SO_4_^2−^. In the presence of foreign components that can affect acidity, it is necessary to adjust the pH up to ≥6.5 by adding alkali to obtain BztCl–NaLS–H_2_O.

### 3.5. Analysis of Real Samples

To demonstrate the applicability of the developed method, the determination of dyes in two commercial beverages, the shell of a pharmaceutical drug, and food-processing industrial wastewater was carried out and compared with the spectrophotometric determination after the preconcentration procedure (Table 2). The results obtained by the smartphone-based colorimetric method are in good agreement with those obtained by the UV–Vis method.

Recovery tests were carried out in beverages and food-processing industrial wastewater (Table 2). In the case of the Allura Red and Azorubine determinations, the found dye content matched the added amount. This indicates the good accuracy of the determination. The relative standard deviation (RSD) values for three measurements were < 6% using the spectrophotometric method and did not exceed 14% using the smartphone-based method, which proves the sufficient reproducibility of both methods. The results obtained by both methods correlated well with each other. Considering the dilution of the initial samples, the dye content in the analyzed beverages was evaluated based on the results of two determination methods. The content of Allura Red in “Bubble″ was 18 ± 3 mg L^−1^ (*t* and *F* values were 1.2 and 2.3, estimated at a 95% confidence level; *t*_critical_ = 2.78, *F*_critical_ = 19.2). The content of Azorubine in “Atom″ was 48 ± 2 mg L^−1^ (*t* and *F* values were 2.1 and 1.6, estimated at a 95% confidence level; *t*_critical_ = 2.78, *F*_critical_ = 19.2). The recovery ranged from 91 to 111%, confirming its good accuracy and applicability.

The results showed that there was no contamination with the studied dyes of food-processing industrial wastewater. To evaluate the accuracy, water samples were spiked with the dye standards at 0.08 and 0.16 mg L^−1^ for Fast Green FCF and 0.10 and 0.50 mg L^−1^ for all other dyes. As can be seen, good recoveries of 95–110% were obtained for the spectrophotometric measurements and 85–112% for the smartphone-based measurements. Therefore, the proposed method provided the merits for the determination of dye residues in water samples.

The proposed method was used to determine Sunset Yellow in the shell of a drug. The value obtained for Sunset Yellow coincides with the data declared by the manufacturer (Table 2).

## 4. Conclusions

A new ATPS-based microextraction coupled with colorimetry was successfully developed to determine synthetic food dyes. The proposed benzethonium-based ATPSs supported the green sample preparation by using a mixture of cationic and anionic surfactants as the extractant. The extraction of dyes was carried out simultaneously with the formation of ATPS by mixing BztCl and NaA (where A = LS or DHSS) at an equimolar ratio. The extraction efficiency was at least 98%. The procedure is favorably characterized by short time, low reagent consumption, and a small amount of residues. In addition, it does not require any organic solvents. The subsequent colorimetric determination does not require the separation of the aqueous phase, and analytical measurements are carried out directly in a test tube containing two phases. The disadvantages of the smartphone-based colorimetric method compared to spectrophotometry are lower sensitivity and a narrower determination range; its advantages are avoiding additional boxes and cuvettes, low cost, easily used the equipment, compactness and mobility, the ability to measure several samples simultaneously, and good integration with the Internet.

## Figures and Tables

**Table 1:** Comparison of the spectrophotometric and smartphone-based colorimetric determination of dyes.

	BztCl–NaLS–H_2_O		BztCl–NaDHSS–H_2_O	
	Spectrophotometry	Colorimetry	Spectrophotometry	Colorimetry
Allura Red AC				
Determination range, mg L^−1^	0.03–2.1	0.1–2.1	0.02–2.1	0.09–2.1
Equation	A = 0.6681*c* − 0.0052	B/R = 0.021 + 0.90exp(−*c*/0.87)	A = 0.9098*c* − 0.0005	B/R = 0.15 + 0.87exp(−*c*/0.40)
R^2^	0.9988	0.9985	0.9997	0.9968
LOD, mg L^−1^	0.009	0.03	0.004	0.03
LOQ, mg L^−1^	0.03	0.1	0.02	0.09
RSD ^a^, %	2	5	1	3
Azorubine				
Determination range, mg L^−1^	0.03–1.6	0.1–1.6	0.02–1.6	0.02–1.6
Equation	A = 0.5887*c* + 0.0363	G/R = 0.25 + 0.72exp(−*c*/0.46)	A = 0.8429*c* + 0.0296	G/R = 0.32 + 0.72exp(−*c*/0.27)
R^2^	0.9955	0.9932	0.9988	0.9942
LOD, mg L^−1^	0.01	0.03	0.005	0.005
LOQ, mg L^−1^	0.03	0.1	0.02	0.02
RSD ^a^, %	2	4	3	5
Sunset Yellow				
Determination range, mg L^−1^	0.03–1.9	0.2–1.5	0.03–1.9	0.04–1.5 0.03–0.4 ^b^
Equation	A = 0.5589*c* + 0.0269	B/R = 0.13 + 0.83exp(−*c*/0.65)	A = 0.7895*c* − 0.0052	B/R = 0.36 + 1.1exp(−*c*/0.23) B/R = −2.3918*c* + 0.9215 ^b^
R^2^	0.9989	0.9928	0.999	0.9938 (0.9955) ^b^
LOD, mg L^−1^	0.01	0.05	0.008	0.01 (0.02) ^b^
LOQ, mg L^−1^	0.04	0.2	0.03	0.04 (0.08) ^b^
RSD ^a^, %	3	5	2	5
Tartrazine				
Determination range, mg L^−1^	0.04–2.3	0.04–2.3	0.02–2.3	0.04–0.7 0.08–0.4 ^b^
Equation	A = 0.6529*c* + 0.0116	B/R = 0.97exp(−*c*/0.50)	A = 0.6744*c* + 0.0444	B/R = 0.07 + 0.89exp(−*c*/0.25) B/R = −1.9834*c* + 0.9276 ^b^
R^2^	0.9995	0.9945	0.9947	0.9948 (0.9904) ^b^
LOD, mg L^−1^	0.009	0.02	0.009	0.01 (0.02)^b^
LOQ, mg L^−1^	0.03	0.06	0.02	0.04 (0.08) ^b^
RSD ^a^, %	2	5	3	10
Fast Green FCF				
Determination range, mg L^−1^	0.01–1.1	0.06–0.6 0.08–0.4 ^b^	0.01–0.7	0.03–0.4 0.04–0.08 ^b^
Equation	A = 1.1247*c* + 0.0099	R/G = −0.45 + 1.47exp(−*c*/0.48) R/G = −2.2054*c* + 1.0013 ^b^	A = 2.4843*c* + 0.0234	R/G = −0.05 + 0.95exp(−*c*/0.13) R/G = −5.5643*c* + 0.9006 ^b^
R^2^	0.9988	0.9965 (0.999) ^b^	0.9955	0.9932 (0.9848) ^b^
LOD, mg L^−1^	0.005	0.02 (0.02) ^b^	0.004	0.008 (0.01) ^b^
LOQ, mg L^−1^	0.02	0.06 (0.08) ^b^	0.01	0.03 (0.04) ^b^
RSD ^c^, %	6	10	4	12

^a^ 0.35 mg L^−1^, *n* = 3; ^b^ Results calculated using the linear approximation; ^c^ 0.08 mg L^−1^, *n* = 3.

**Table 2:** Spectrophotometric and smartphone-based colorimetric determination of dyes in samples after preconcentration in BztCl–NaDHSS-H_2_O (*n* = 3, *P* = 0.95).

		Spectrophotometric Determination			Smartphone-Based Colorimetric Determination		
Sample–Dye	Added, mg L^−1^	Found, mg L^−1^	RSD, %	Recovery, %	Found, mg L^−1^	RSD, %	Recovery, %
“Bubble″ beverage–Allura Red	0	0.17 ± 0.02	5	–	0.18 ± 0.03	2	–
	0.21	0.37 ± 0.04	4	95	0.38 ± 0.03	3	95
	0.42	0.61 ± 0.02	1	105	0.65 ± 0.05	3	111
“Atom″ beverage –Azorubine	0	0.47 ± 0.04	3	–	0.50 ± 0.05	4	–
	0.16	0.64 ± 0.05	3	106	0.65 ± 0.10	6	94
	0.33	0.81 ± 0.04	2	103	0.80 ± 0.12	7	91
Shell of the drug “Nitroxalin″– Sunset Yellow ^a^	0	0.070 ± 0.007 ^b^	4	–	0.075 ± 0.01	6	–
Wastewater– Allura Red	0	n.d. ^c^	–	–	n.d. ^c^	–	–
	0.10	0.11 ± 0.02	6	110	0.11 ± 0.03	11	110
	0.50	0.48 ± 0.04	3	96	0.46 ± 0.08	7	92
Wastewater– Azorubine	0	n.d. ^c^	–	–	n.d. ^c^	–	–
	0.10	0.095 ± 0.010	4	95	0.085 ± 0.005	3	85
	0.50	0.50 ± 0.05	4	100	0.52 ± 0.10	7	104
Wastewater– Sunset Yellow	0	n.d. ^c^	–	–	n.d. ^c^	–	–
	0.10	0.10 ± 0.01	4	100	0.11 ± 0.04	14	110
	0.50	0.52 ± 0.04	3	104	0.55 ± 0.05	4	110
Wastewater– Tartrazine	0	n.d. ^c^	–	–	n.d. ^c^	–	–
	0.10	0.095 ± 0.005	2	95	0.085 ± 0.02	10	85
	0.50	0.52 ± 0.04	3	104	0.45 ± 0.10	9	90
Wastewater– Fast Green FCF	0	n.d. ^c^	–	–	n.d. ^c^	–	–
	0.08	0.085 ± 0.010	4	106	0.09 ± 0.02	9	112
	0.16	0.16 ± 0.02	5	100	0.17 ± 0.02	5	106

^a^ Label claim/manufacturer’s data: 0.07 mg; ^b^ mg; ^c^ Not detected.

## Data Availability

The data presented in this study are available on request from the corresponding author.

## Supplementary Materials

The following supporting information can be downloaded at: https://www.mdpi.com/article/10.3390/s23073519/s1, Table S1: Data for plotting the BztCl–NaLS–H_2_O diagram; Table S2: Data for plotting the BztCl–NaDHSS–H_2_O diagram.
