# Supplementary material for: Aqueous Two-Phase Systems Based on Cationic and Anionic Surfactants Mixture for Rapid Extraction and Colorimetric Determination of Synthetic Food Dyes
Source: Sensors (Basel). 2023 Mar 28;23(7):3519. doi: 10.3390/s23073519 (PMC10099167; doi:10.3390/s23073519)
Supplement: Supplementary file 1 [file sensors-23-03519-s001.zip › sensors-2296257-supplementary.pdf]

Supplementary Materials

Table of contents

Table S1. Data for plotting the BztCl–NaLS–H_2O diagram (titration results).

Table S2. Data for plotting the BztCl–NaDHSS–H_2O diagram.

Table S1. Data for plotting the BztCl–NaLS–H_2O diagram (titration results).

Stock solutions $c_{BztCl} = c_{NaLS} = 0.2$ mol L^{-1}			Titrant (BztCl) volume, mL	c_{BztCl}, mol L^{-1}	c_{NaLS}, mol L^{-1}
V_{BztCl}, mL	V_{NaLS}, mL	V_{H2O}, mL			
1.0	1.0	0	1.50	0.1429	0.0571
1.0	1.0	0,5	1.40	0.1231	0.0513
1.0	1.0	1	1.27	0.1063	0.0468
1.0	1.0	2	1.03	0.0807	0.0398
1.0	1.0	3	0.96	0.0658	0.0336
1.5	1.5	6	1.33	0.0548	0.0290
1.5	1.5	9	1.16	0.0404	0.0228
1.5	1.5	12	1.05	0.0318	0.0187
1.5	1.5	18	0.80	0.0211	0.0138
1.5	1.5	27	0.63	0.0139	0.0098
1.5	1.5	57	0.54	0.0067	0.0050
			Titrant (NaLS) volume, mL		
1.5	1.5	0	1.15	0.0723	0.1277
1.5	1.5	0.75	1.14	0.0613	0.1080
1.5	1.5	1.5	1.08	0.0538	0.0925
1.5	1.5	3	1.00	0.0429	0.0714
1.5	1.5	6	0.96	0.0301	0.0494
1.5	1.5	9	0.89	0.0233	0.0371
2.0	2.0	16	1.15	0.0189	0.0298
2.0	2.0	24	1.03	0.0138	0.0209
2.0	2.0	40	1.01	0.0089	0.0134
2.0	2.0	80	1.39	0.0047	0.0079

Table S2. Data for plotting the BztCl–NaDHSS–H_2O diagram.

№	Concentration of stock solutions, mol L^{-1}		Volume of stock solution[a], mL		Concentration, mol L^{-1}		Phase Behavior BztCl/NaDHSS mixtures[b]
	c_{BztCl}	c_{NaDHSS}	BztCl	NaDHSS	NaDHSS	BztCl	
Series 1	0.200	0.200	2.0	2.0	0.1000	0.1000	L1 + L2
	0.200	0.200	1.5	1.5	0.0750	0.0750	L1 + L2
	0.200	0.200	1.0	1.0	0.0500	0.0500	L1 + L2
	0.200	0.200	0.8	0.8	0.0400	0.0400	L1 + L2
	0.200	0.200	0.7	0.7	0.0350	0.0350	L1 + L2
	0.200	0.200	0.5	0.5	0.0250	0.0250	L1 + L2
	0.200	0.200	0.4	0.4	0.0200	0.0200	L1 + L2
	0.200	0.200	0.35	0.35	0.0175	0.0175	L1 + L2
	0.200	0.200	0.3	0.3	0.0150	0.0150	L1 + L2
	0.200	0.200	0.2	0.2	0.0100	0.0100	L1 + L2
	0.200	0.200	0.15	0.15	0.0055	0.0055	L1 + L2
	0.200	0.200	0.1	0.1	0.0050	0.0050	L1 + L2
Series 2	0.1464	0.1464	0.2	0.2	0.0073	0.0073	L1 + L2
	0.1464	0.1464	0.25	0.25	0.0092	0.0092	L1 + L2
	0.1464	0.1464	0.3	0.3	0.0110	0.0110	L1 + L2
	0.1464	0.1464	0.5	0.5	0.0183	0.0183	L1 + L2
	0.1464	0.1464	2.0	2.0	0.0732	0.0732	L1 + L2
Series 3	0.200	0.200	0.02	3.0	0.1500	0.0010	L
	0.200	0.200	0.05	3.0	0.1500	0.0025	L
	0.200	0.200	0.1	3.0	0.1500	0.0050	L
	0.200	0.200	0.2	3.0	0.1500	0.0100	L+ S
	0.200	0.200	1.0	3.0	0.1500	0.0500	L+ S
Series 4	0.1464	0.1464	0.03	1.0	0.0366	0.0001	L
	0.1464	0.1464	0.03	1.0	0.0366	0.0011	L
	0.1464	0.1464	0.3	1.0	0.0366	0.011	L+ S
	0.1464	0.1464	0.6	1.0	0.0366	0.0220	L+ S
	0.1464	0.1464	1.0	1.0	0.0366	0.0366	L1 + L2
	0.1464	0.1464	1.4	1.0	0.0366	0.0512	L1 + L2
	0.1464	0.1464	2.0	1.0	0.0366	0.0732	L+ S
	0.1464	0.1464	2.7	1.0	0.0366	0.09882	L+ S
Series 5	0.1464	0.1464	0.03	1.4	0.0512	0.0011	L
	0.1464	0.1464	0.3	1.4	0.0512	0.0109	L
	0.1464	0.1464	0.6	1.4	0.0512	0.0220	L+ S
	0.1464	0.1464	1.0	1.4	0.0512	0.0366	L+ S
	0.1464	0.1464	1.4	1.4	0.0512	0.0512	L1 + L2
	0.1464	0.1464	2.0	1.4	0.0512	0,0732	L1 + L2
	0.1464	0.1464	2.6	1.4	0.0512	0.09516	L+ S
Series 6	0.1464	0.1464	0.03	1.5	0.0549	0.0011	L
	0.1464	0.1464	0.15	1.5	0.0549	0.0055	L+ S
	0.1464	0.1464	0.27	1.5	0.0549	0.0099	L+ S
	0.1464	0.1464	1.5	1.5	0.0549	0.0549	L1 + L2
	0.1464	0.1464	2.5	1.5	0.0549	0.0915	L+ S

Series 7	0.1464	0.1464	0.15	2.0	0.0732	0.0055	L+ S
	0.1464	0.1464	0.27	2.0	0.0732	0.0099	L+ S
	0.1464	0.1464	0.8	2.0	0.0732	0.0293	L+ S
	0.1464	0.1464	1.4	2.0	0.0732	0.0512	L+ S
	0.1464	0.1464	2.0	2.0	0.0732	0.0732	L1 + L2
	0.1464	0.1464	2.0	2.0	0.0732	0.1000	L+ S
Series 8	0.1464	0.1464	0.03	0.55	0.0202	0.0001	L+ S
	0.1464	0.1464	0.013	0.55	0.0202	0.0005	L+ S
	0.1464	0.1464	0.03	0.55	0.0202	0.0011	L+ S
	0.1464	0.1464	0.15	0.55	0.0202	0.0055	L+ S
	0.1464	0.1464	0.27	0.55	0.0202	0.0099	L+ S
	0.1464	0.1464	1.0	0.55	0.0202	0.0366	L+ S
	0.1464	0.1464	1.9	0.55	0.0202	0.0695	L+ S
	0.1464	0.1464	2.73	0.55	0.0202	0.0999	L+ S
	0.1464	0.1464	0.71	0.55	0.0202	0.0260	L1 + L2
	0.1464	0.1464	0.55	0.55	0.0202	0.0200	L1 + L2
Series 9	0.1464	0.1464	0.27	2.7	0.0988	0.0099	L+ S
	0.1464	0.1464	0.8	2.7	0.0988	0.0293	L+ S
	0.1464	0.1464	1.4	2.0	0.1000	0.0512	L+ S
	0.1464	0.1464	2.0	2.0	0.1000	0.0732	L1 + L2
	0.1464	0.1464	2.0	2.0	0.1000	0.1000	L1 + L2
Series 10	0.1464	0.1464	0.7	0.75	0.0275	0.0256	L1 + L2
	0.1464	0.1464	0.75	0.75	0.0275	0.0275	L1 + L2
	0.1464	0.1464	0.8	0.75	0.0275	0.0293	L1 + L2
	0.1464	0.1464	0.9	0.75	0.0275	0.0329	L1 + L2
	0.1464	0.1464	1.2	0.75	0.0275	0.0439	L+ S
Series 11	0.200	0.200	0.25	0.3	0.0150	0.0125	L+ S
	0.200	0.200	0.3	0.3	0.0150	0.0150	L1 + L2
	0.200	0.200	0.4	0.3	0.0150	0.0200	L1 + L2
	0.200	0.200	0.36	0.3	0.0150	0.0180	L1 + L2
Series 12	0.200	0.200	0.35	0.35	0.0175	0.0175	L1 + L2
	0.200	0.200	0.3	0.35	0.0175	0.015	L+ S
	0.200	0.200	0.4	0.35	0.0175	0.0200	L1 + L2
	0.200	0.200	0.45	0.35	0.0175	0.0225	L+ S
	0.200	0.200	0.37	0.35	0.0175	0.0185	L1 + L2
	0.200	0.200	0.42	0.35	0.0175	0.0210	L1 + L2
Series 13	0.200	0.200	0.35	0.45	0.0225	0.0175	L+ S
	0.200	0.200	0.4	0.45	0.0225	0.0200	L1 + L2
	0.200	0.200	0.45	0.45	0.0225	0.2250	L1 + L2
	0.200	0.200	0.5	0.45	0.0225	0.0250	L1 + L2
	0.200	0.200	0.6	0.45	0.0225	0.0300	L+ S
Series 14	0.200	0.200	0.9	1.25	0.0625	0.0450	L+ S
	0.200	0.200	1.1	1.25	0.0625	0.0550	L1 + L2
	0.200	0.200	1.25	1.25	0.0625	0.06250	L1 + L2
	0.200	0.200	1.4	1.25	0.0625	0.0700	L1 + L2
	0.200	0.200	1.6	1.25	0.0625	0.0800	L1 + L2

Series 15	0.1464	0.1464	0.7	0.85	0.0311	0.0256	L+ S
	0.1464	0.1464	0.8	0.85	0.0311	0.0293	L1 + L2
	0.1464	0.1464	0.9	0.85	0.0311	0.0329	L1 + L2
	0.1464	0.1464	1.1	0.85	0.0311	0.0403	L1 + L2
	0.1464	0.1464	1.3	0.85	0.0311	0.0476	L+ S
Series 16	0.1464	0.1464	1.0	1.2	0.0439	0.0366	L+ S
	0.1464	0.1464	1.1	1.2	0.0439	0.0404	L1 + L2
	0.1464	0.1464	1.3	1.2	0.0439	0.0477	L1 + L2
	0.1464	0.1464	1.5	1.2	0.0439	0.0549	L1 + L2
	0.1464	0.1464	1.7	1.2	0.0439	0.0622	L+ S
Series 17	0.200	0.200	1.4	1.75	0.0875	0.0700	L1 + L2
	0.200	0.200	1.5	1.75	0.0875	0.0750	L1 + L2
	0.200	0.200	1.7	1.75	0.0875	0.0850	L1 + L2
	0.200	0.200	1.85	1.75	0.0875	0.0920	L1 + L2
	0.200	0.200	2.0	1.75	0.0875	0.1000	L+ S
Series 18	m_{BztCl}, g	m_{NaDHSS}, g	m_{H2O}, g		c_{BztCl}, mol L^{-1}	c_{NaDHSS}, mol L^{-1}	
	0.1777	0.2174	3.60		0.0993	0.1401	L1 + L2
	0.1735	0.2467	3.69		0.0943	0.1545	L1 + L2
	0.1551	0.1527	3.69		0.0864	0.0983	L1 + L2
	0.1558	0.1858	3.65		0.0872	0.1198	L1 + L2
	0.1315	0.1694	3.70		0.0734	0.1090	L1 + L2
	0.1831	0.1868	3.63		0.1022	0.1202	L1 + L2
	0.1777	0.2174	3.60		0.0993	0.1401	L1 + L2
	0.1735	0.2467	3.69		0.0942	0.1545	L1 + L2
	0.1551	0.1527	3.69		0.0866	0.0983	L1 + L2
	0.1558	0.1858	3.65		0.0871	0.1198	L1 + L2
	0.1315	0.1694	3.70		0.0734	0.1090	L1 + L2
	0.1768	0.1333	3.69		0.0987	0.0858	L1 + L2
	0.0986	0.0727	1.83		0.1100	0.0936	L1 + L2
	0.1757	0.1384	3.69		0.0980	0.0890	L1 + L2
	0.0986	0.0959	1.80		0.1102	0.1234	L1 + L2

[a] The final volume for each sample of the series was 4.0 ml (diluted with water)

[b] $L_1 + L_2$ – the ATPS region; L + S – the heterogeneous system with a precipitate; L – one liquid phase
